# C-Myc functions as a competing endogenous RNA in acute promyelocytic leukemia

**DOI:** 10.18632/oncotarget.10896

**Published:** 2016-07-28

**Authors:** Ye Ding, Ze-chuan Wang, Yi Zheng, Zheng Hu, Yang Li, Dong-feng Luo, Shao-yuan Wang

**Affiliations:** ^1^ Union Clinical Medical College, Fujian Medical University, Fuzhou, P.R. China; ^2^ Department of Hematology, Fujian Institute of Hematology, Fujian Provincial Key Laboratory on Hematology, Fujian Medical University Union Hospital, Fuzhou, P.R. China

**Keywords:** ceRNA, APL, Let-7, Myc, PML/RARα

## Abstract

Recent reports have described a new post-transcriptional regulation that RNA transcripts can crosstalk with each other by competing for their common microRNAs. These RNA transcripts termed competing endogenous RNAs (ceRNAs) regulate the distribution of miRNAs on their targets. One corollary from ceRNA interaction is that chromosomal translocation in acute promyelocytic leukemia (APL) would perturb ceRNA regulation due to altered expression of 3′UTRs. In our study, we demonstrate that expression of PML/RARα, the APL-associated fusion oncogene is repressed by c-Myc mRNA transcript independent of protein-coding function but dependent upon microRNA. Attenuation of c-Myc transcript results in PML/RARα-degraded cellular phenotypes in APL cells, but these Myc reduction-associated cell phenotypes are sufficient to abrogate in a microRNA dependent manner. We also show that let-7 microRNA family members promote differentiation of All-Trans-Retinoic Acid (ATRA)-induced NB4 cells and their activities are affected by expression levels of both c-Myc and PML/RARα through altering miRNA targets. These results indicate that c-Myc mRNA represses PML/RARα expression via altering the distribution of let-7 miRNAs on their targets. Our findings reveal a previously unrecognized role of c-Myc as a potential ceRNA for PML/RARα in APL.

## INTRODUCTION

MicroRNAs (miRNAs) are small non-coding RNAs of approximately 22 nucleotides in length that direct degradation of target RNA transcripts or inhibition of its translation in a sequence-dependent manner [1]. Control of gene expression by miRNAs is critical for maintaining a variety of important biological processes, including development, differentiation, and hematopoiesis [2, 3]. Despite growing knowledge on miRNA biology, little is known about how and if mRNAs can escape regulation by a miRNA [4]. A recently discovered mechanism for post-transcriptional regulation has been described that the activity of miRNA is regulated by the abundance of their targets in a sequence-specific manner, and as a consequence, the concentration of a target RNA may impact the activity of a miRNA on its other targets, thus, these RNA targets (ceRNAs) act as miRNA decoys and co-regulate with each other by competing for shared miRNAs [5–8]. It were further speculated that the ceRNA activity could be perturbed by chromosomal translocation which often occurred in multiple subtypes of leukemia, such as acute promyelocytic leukemia (APL), a specific chromosomal translocation *t* (15;17) involving the promyelocytic leukemia (PML) gene locus on chromosome 15 and the retinoid acid receptor alpha (RARα) gene locus on chromosome 17. Such event could be considered ‘UTR swaps’, leading to changes in the expression of UTRs and consequent altering the competition of miRNAs [6]. But there are not experimental evidences to confirm that so far.

The proto-oncogene c-Myc is known to promote cell growth and proliferation and plays pivotal for maintenance of the cell cycle in most hematopoietic cell lines including granulocytes [9]. In many human hematopoietic malignancies, high c-Myc expression has been correlated with a poor prognosis [10]. In APL, c-Myc is identified as a downstream target of PML/RARα and is frequently activated by PML/RARα via different but less well-understood mechanisms [11–13]. The data accumulated in cell and animal models indicated that down-regulation of c-Myc expression allows myeloid cells to enter into the granulocytic differentiation pathway [12]. Failure to down-regulate c-Myc in transgenic mice, can lead to myeloid leukemia, a condition characterized by a block in differentiation. These suggest inhibition of c-Myc is a critical event for a cell to commit to a differentiation pathway [14, 15]. In short, Myc can exhibit opposite cellular functions on unrestrained proliferation and differentiation. As proliferation and differentiation are mutually exclusive, we wonder if there exists a switch which controls cell proliferation and differentiation via altering c-Myc expression.

The let-7 miRNA family members control the timing of cell cycle exit and terminal differentiation in Caenorhabditis elegans [16, 17]. In humans, there are 12 let-7 family members (let-7a-1, −2, −3; let-7b; let-7c; let-7d; let-7e; let-7f-1, −2; let-7g; let-7i; miR-98) located at eight different chromosomal loci. Of note, let-7 miRNAs directly or indirectly repress multiple cell cycle oncogenes such as Myc, Ras, HMGA2, and lin28B [18–24], these strongly suggest that let-7 is a key regulator of cell cycle progression and differentiation.

The fusion oncogene PML/RARα causes differentiation arrest at the promyelocytic stage of development [25, 26]. MiR target prediction indicated that PML/RARα is one of the putative target genes of let-7 family members. Additionally, let-7c has been reported to display lower levels in PML/RARα-positive blasts from APL patients than normal promyelocytes and promote granulocytic differentiation of AML cell lines and primary blasts [27].

Based on the above knowledge, it could be speculated that let-7 family members probably would play a pivotal role through modulating the levels of c-Myc and other let-7-responsive genes to regulate the exquisite balance between differentiation and proliferation required for blood formation. Ectopic expression of PML/RARα in APL would destroy this balance and transform hematopoietic cells by stimulating proliferation and blocking terminal differentiation. In the present study, we extend the ceRNA hypothesis to APL which is induced by misplacement to investigate the effect of altered c-Myc transcript on PML/RARα from novel perspective and conclude that Myc mRNA transcript functions as a potential ceRNA for PML/RARα by competing for let-7 miRNAs.

## RESULTS

### Repression of PML/RARα by c-Myc loss is miRNA dependent

**Figure 1:**
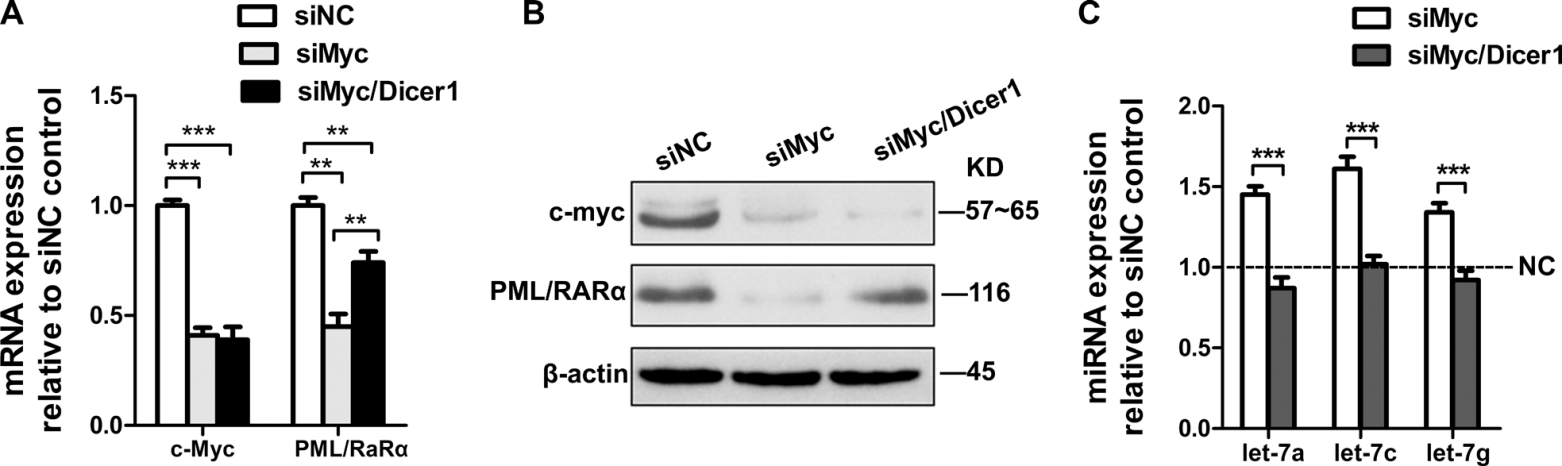
Repression of PML/RARα by c-Myc is miRNA dependent (**A**) QRT-PCR analysis of c-myc and PML/RARα mRNA changes at 24 hr following siRNA mediated silencing of c-myc with and without siRNA-mediated silencing of Dicer1. The data were normalized to nontarget siRNA (siNC) transfection. (**B**) Western blot showing c-myc and PML/RARα protein expression 48 h after transfection of the indicated siRNAs. (**C**) MicroRNA RT-PCR showing expression of let-7a, 7c and 7g in response to siRNA against c-myc alone or together with siRNA against Dicer1. siNC was used as a control. Bar graph was represented as mean ± SD of three independent experiments performed in triplicates (*n* = 3). ** indicated *P* < 0.01 and *** indicated *P* < 0.001. Representative blot was shown from three independent experiments.

We first investigated the ability of c-Myc mRNA to modulate PML/RARα levels by examining the effect of depletion of c-Myc on endogenous PML/RARα levels. Silencer^®^ Select siRNA was used in our experiment, which is designed to achieve strong on-target gene knockdown by lower concentration with minimal off-target effects. We transfected NB4 cells (APL cell lines) transiently with siRNA for Myc (the negative control transfection at the same time points). Real-time PCR analysis confirmed the efficiency of siRNA-mediated gene knockdown. As shown in Figure 1A and 1B, depletion of Myc resulted in the reduction of PML/RARα mRNA and protein levels. To ascertain whether PML/RARα reduction by Myc loss is in a microRNA-dependent manner, we transfected transiently Myc siRNA alone or together with Dicer1 siRNA into NB4 cells. As Dicer1 is a critical enzyme involved in the processing of mature microRNAs, the Dicer1 deficient cells present an ideal system to evaluate microRNA-dependent effects. QRT-PCR confirmed the efficient lowered expression of mature let-7 miRNAs by Dicer1 knockdown (Figure 1C). We found that siRNA-mediated silencing of Dicer1 rescued the effects of Myc knockdown on PML/RARα repression in both mRNA and protein levels (Figure 1A and 1B), suggesting that reduction of PML/RARα expression by Myc knockdown is microRNA dependent.

### Tumor-suppressive properties mediated by c-Myc silencing are attenuated when Dicer1 expression is abrogated

**Figure 2:**
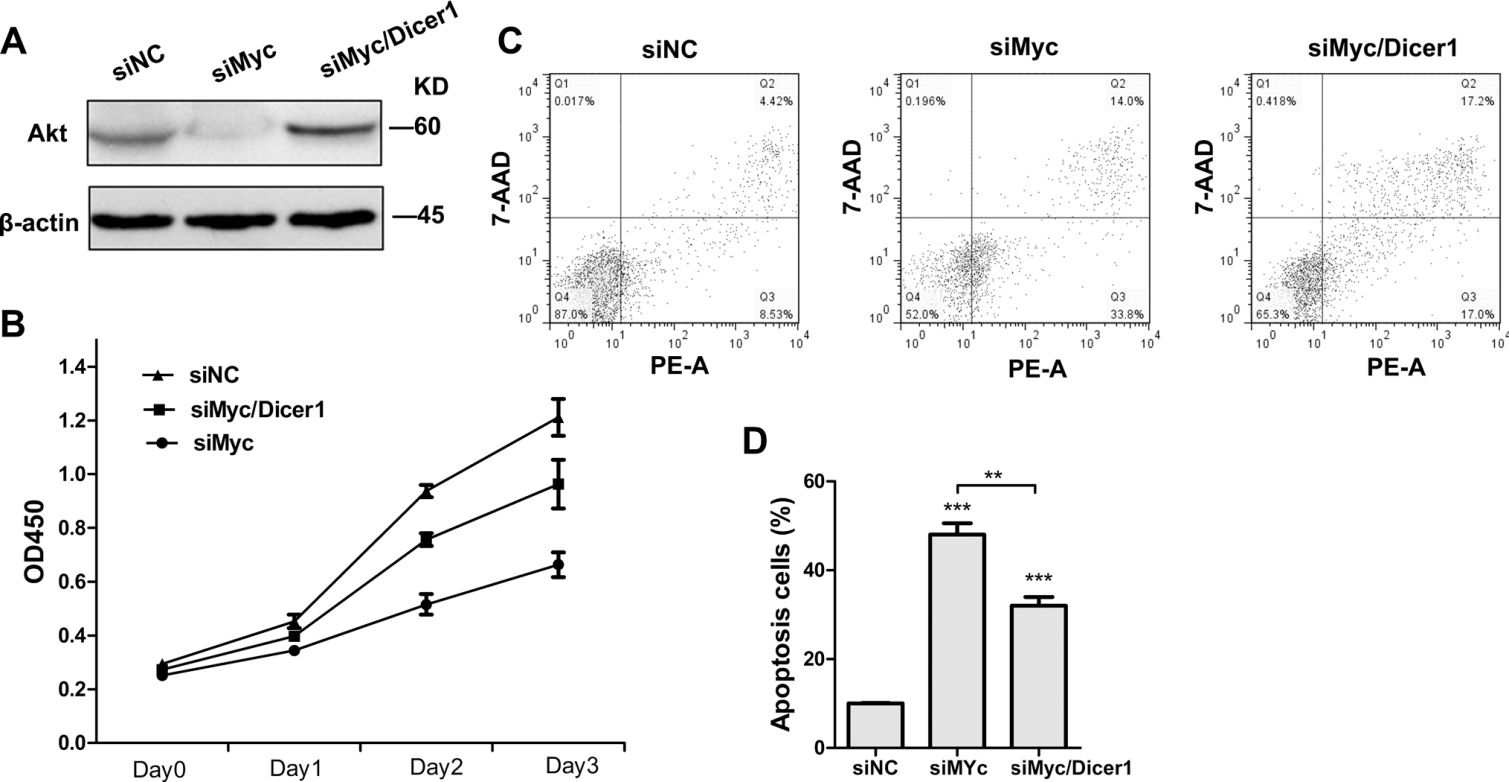
Depletion of Dicer1 abrogated the effect of c-Myc silencing in NB4 cells (**A**) Western blot of total AKT 48 h after the transfection with siRNAs agaist c-myc alone (siMyc) and together with siRNA against Dicer1 (siMyc/Dicer1). (**B**) Cell proliferation curve of NB4 cells transfected with the indicated siRNAs (*n* = 3, *p* < 0.05). (**C**) Apoptosis of NB4 cells treated with siNC, siMyc and siMyc/Dicer1 for 72 h as assessed by flow cytometry. PE-A on X-axis stands for PE-Annexin V. (**D**) Quantification of apoptosis cells shown in (C). Representative blot was shown from three independent experiments. Bar graph was represented as mean ± SD of three independent experiments performed in triplicates (*n* = 3). ** indicated *P* < 0.01 and *** indicated *P* < 0.001.

According to ceRNA function that a ceRNA is co-regulated with its target gene, we speculated that the effects of Myc mRNA ablation on APL cells are similar to that of PML/RARα degradation, which is triggered by Arsenic Trioxide (ATO, a highly effective anti-leukemic agent) and consequently results in APL cell apoptosis [28, 29]. Therefore, we first explored the consequences of Myc depletion on AKT protein as well as cell proliferation and apoptosis. As expected, Western blot confirmed that siMyc inhibited AKT protein levels in NB4 cells compared to those transfected with control siRNA (siNC) (Figure 2A). Furthermore, reduced Myc expression by siRNA resulted in a decrease in proliferation (Figure 2B) and increase in the percentage of apoptotic cell (Figure 2C and 2D), consistent with the phenotype of degraded PML/RARα protein in NB4 cells. Nevertheless, the above observed phenotype was to some extent abrogated, when NB4 cells were transfected with siMyc together with siDicer1 (Figure 2A–2D). These results strongly suggest that Myc-induced PML/RARα repression in NB4 cells is at least partially in a microRNA-dependent manner.

### Let-7 family members promote granulocytic differentiation

**Figure 3:**
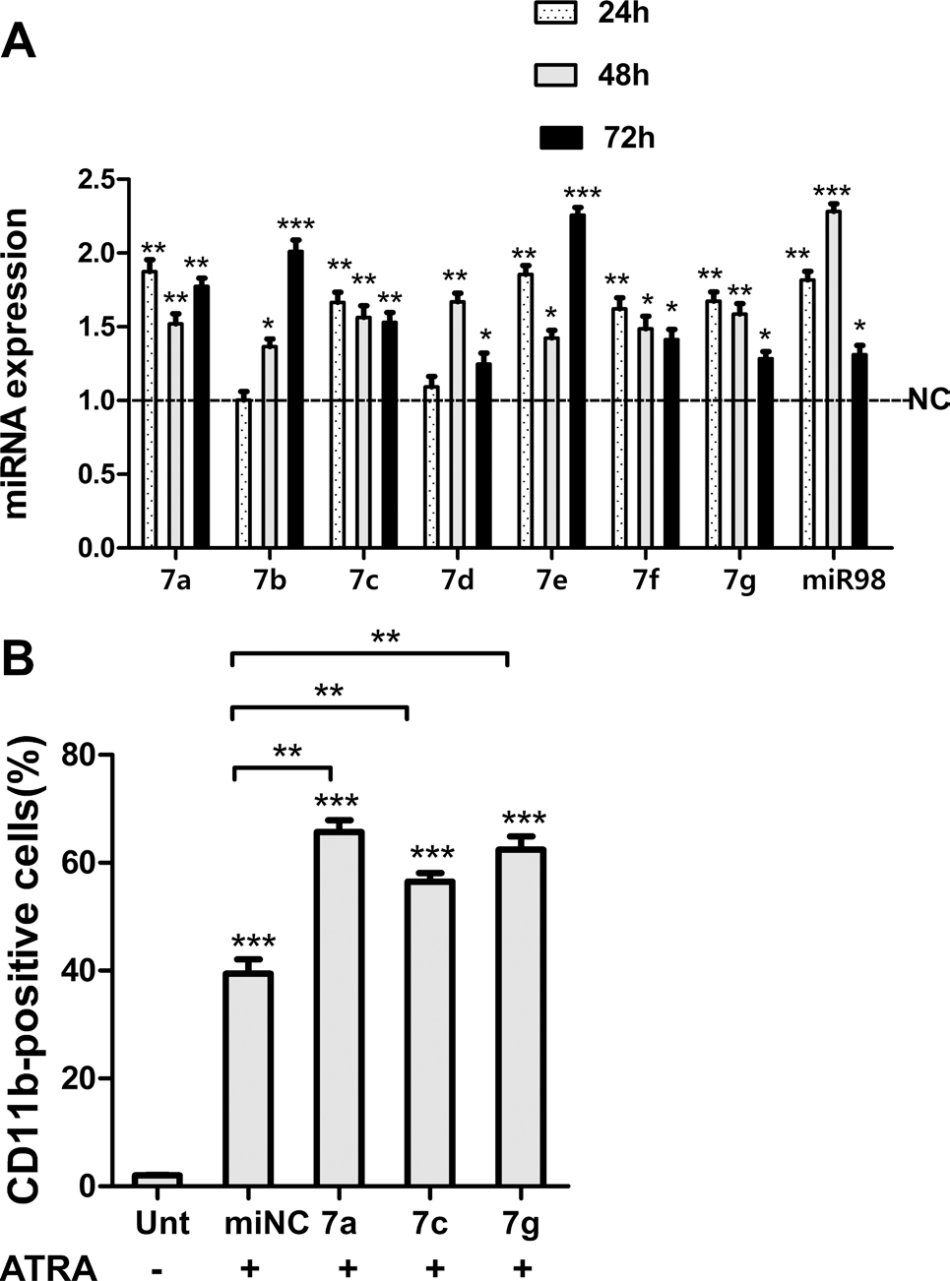
Let-7 miRNAs promoted granulocytic differentiation of NB4 cells (**A**) RNA levels of indicated let-7 family members in NB4 cells treated with ATRA for 24, 48 and 72 h respectively, values were compared with DMSO-treated control cells (NC) in corresponding times. (**B**) CD11b expression assessed by flow cytometry in ATRA-treated cells (12 h after transfection) at 48 hr following ectopic expression of indicated let-7 miRNAs. Data were represented as mean ± SD of three independent experiments in triplicates (*n* = 3). * indicated *P* < 0.05, ** indicated *P* < 0.01 and *** indicated *P* < 0.001.

Low expression of let-7 family members correlates with increased transformation capacity *in vitro* and poor survival *in vivo* [30]. Of note, PML/RARα positive blasts from APL patients display lower levels of miRNA let-7c than normal promyelocytes [27]. Herein we investigated whether let-7 family members contribute to granulocytic differentiation of NB4 cell lines. First, we determined the differential expression levels of let-7 miRNA in NB4 cells with or without All-Trans-Retinoic Acid (ATRA, differentiating agent for APL-treated) treatment. To address this, NB4 cells were treated with 1 μM ATRA or Dimethyl Sulfoxide (DMSO, negative control) for 24, 48, 72 and 96 hr respectively. MiRNA specific qRT-PCR was performed to analyze the expression of let-7 family (miR-let-7a,7b,7c,7d,7e,7f,7g and miR-98). QRT-PCR confirmed let-7 members were upregulated upon ATRA treatment compared to DMSO treated cells (control) (Figure 3A). To further investigate the role of let-7 miRNA in APL differentiation, we transfected selectively let-7 mimics (let-7a, 7c, 7g or negative control) into ATRA-treated NB4 cells. We then examined the levels of the α-integrin CD11b, a membrane antigen associated with granulocytic differentiation. We observed the higher levels of CD11b after transfection of let-7 mimics relative to negative control RNA (Figure 3B). Taken together, these data indicated that let-7 miRNAs promote granulocytic differentiation in ATRA-induced NB4 cells.

### Repression of PML/RARα by c-Myc loss is 3′UTR and miRNA dependent

**Figure 4:**
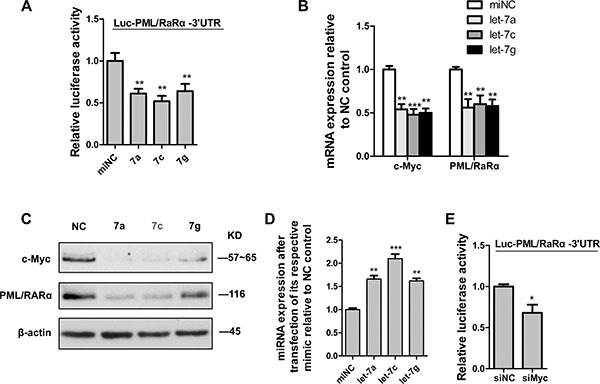
C-Myc suppressed PML/RARα by let-7 miRNAs via 3′UTR (**A**) Luciferase activity in HEK293T cells cotransfected with PML/RARα3′UTR reporter construct and let-7 mimics (7a, 7c and 7g respectively) or scrambled microRNA control (miNC). (**B**) QRT-PCR analysis of c-myc and PML/RARα expression 48 hr after transfection of NB4 cells with let-7 mimics (let-7a, 7c and 7g respectively) and scrambled microRNA control (miNC). (**C**) Western blot analysis for c-myc and PML/RARα 48 hr after transfection of indicated mimics. (**D**) Efficiency of indicated let-7 ectopic expression, measured by qRT-PCR analysis. (**E**) Luciferase activity in HEK293T cells cotransfected with PML/RARα3′UTR reporter construct and siRNA against c-myc or nontarget control (siNC). Bar graph was represented as mean ± SD of three independent experiments performed in triplicates (*n* = 3). * indicated *P* < 0.05, ** indicated *P* < 0.01 and *** indicated *P* < 0.001. Representative blot was shown from three independent experiments.

We have shown that let-7 promoted granulocytic differentiation of APL cell lines. To determine whether the repression of PML/RARα by Myc loss is driven by let-7 sites in their 3′UTRs, we constructed a luciferase construct tagged with the PML/RARα 3′UTR fragment (Luc-PML/RARα3′UTR). Luciferase assays revealed that ectopic expression of miR-let-7a, 7c and 7g respectively reduced Luc-PML/RARα3′UTR activity in 293T cells (Figure 4A). Consistent with the luciferase results, overexpression of let-7a and 7c in NB4 cells resulted in a significant reduction in PML/RARα mRNA levels as well as protein levels, whereas enforced expression of let-7g only decreased PML/RARα mRNA, it did not have a significant effect on PML/RARα protein (Figure 4B and 4C). We surmise that let-7g could probably regulate PML/RARα protein levels independently of its 3′UTR, perhaps via positive modulation of upstream regulators. Additionally, overexpression of let-7a, 7c and 7g respectively caused a significant reduction of c-Myc in mRNA and protein levels in NB4 cells (Figure 4B and 4C), it consists with the previous study on Myc as a direct let-7 target. The efficiency of miRNA mimic overexpression was confirmed by real-time PCR analysis (Figure 4D). These data indicated that let-7 represses Myc or PML/RARα is based on directly binding to their 3′UTRs. Furthermore, siRNA-mediated knockdown of c-Myc transcript lowered Luc-PML/RARα3′UTR activity in 293T cells as a result of increased availability of common miRNAs released from 3′UTR by c-Myc silencing (Figure 4E). All the above data indicated that c-Myc3′UTR functions as a decoy of PML/RARα-targeting miRNA and inhibits PML/RARα expression via altering the distribution of let-7 miRNA on their targets.

## DISCUSSION

A recently proposed mechanism for miRNA has been described that RNA transcripts(ceRNA) can regulate one another through their ability to compete for miRNA binding sites [6]. The gene with perhaps the most extensively characterized ceRNA network is the important tumor suppressor PTEN. Multiple candidates for PTEN ceRNAs by bioinformatics prediction including PTEN pseudogene transcript PTENP1, VAPA, CONT6L, and so on, have been validated experimentally in human cancers [5, 31–33]. Most recently, it was reported that lncRNA-BGL3, a long noncoding RNA which has been identified as a ceRNA for PTEN to regulate Bcr-Abl-mediated cellular transformation in chronic myeloid leukemia [34].

In our study, we confirm that depletion of c-Myc transcript decreased PML/RARα mRNA and protein expression in a Dicer-dependent manner, suggesting mature microRNAs are essential for the regulation of PML/RARα. Furthermore, the effects of c-Myc depletion on AKT attenuation concomitant with inhibition of cell growth and promotion of apoptosis were at least in part abrogated in APL cells of siRNA-mediated reduction of Dicer transcript. These above findings strongly indicate that down-regulation of PML/RARα expression by diminished c-Myc is indeed miRNA-dependent.

Since Myc has been reported to be a direct target of let-7 [35], we then used RNA22, with its low rate of false prediction [36], to predict putative let-7-binding sites in PML/RaRα3′UTR. As the seed sequences in mature let-7 family members are identical, both c-Myc and PML/RARα are highly putative targets of all let-7 family members. We showed that ectopic expression of let-7 reduced both c-Myc and PML/RARα expression in APL cells. On the other hand, the relative luciferase activity of the reporter with wild-type 3′UTR of PML/RARα was suppressed upon co-transfection with mimic let-7. These indicate that let-7 reduces c-Myc and PML/RARα expression is based on a standard interaction of the microRNA with the 3′UTR of the target mRNAs. Furthermore, we find that the decreased Myc transcript in Luc- PML/RARα3′UTR-expressing cells lowered luciferase activity, allowing us to conclude that the ablation of c-Myc mRNA reduces PML/RARα expression is at least partly due to increased availability of common miRNAs.

We also show that let-7 was increased upon ATRA-induced granulocytic differentiation of APL cells, and further promoted APL cell differentiation in combination with ATRA. These data suggest that let-7 family may have a role in APL pathogenesis. Intriguingly, let-7 family members exert dual functions on cell differentiation as well as proliferation by targeting cell cycle progression genes such as Myc, Ras, HMGA2 to control cell-fated decisions [16, 19, 22, 23, 30, 37]. As most of let-7-responsive genes are known as oncogenes, one prediction is that in cancer cells with poor let-7 expression, many of these genes would be up-regulated, which is likely to stimulate cell cycle [16]. Therefore, it may be interesting to investigate how these let-7 target mRNAs control the converted switch between cell differentiation and proliferation by altered competition for shared miRNAs.

**Figure 5:**
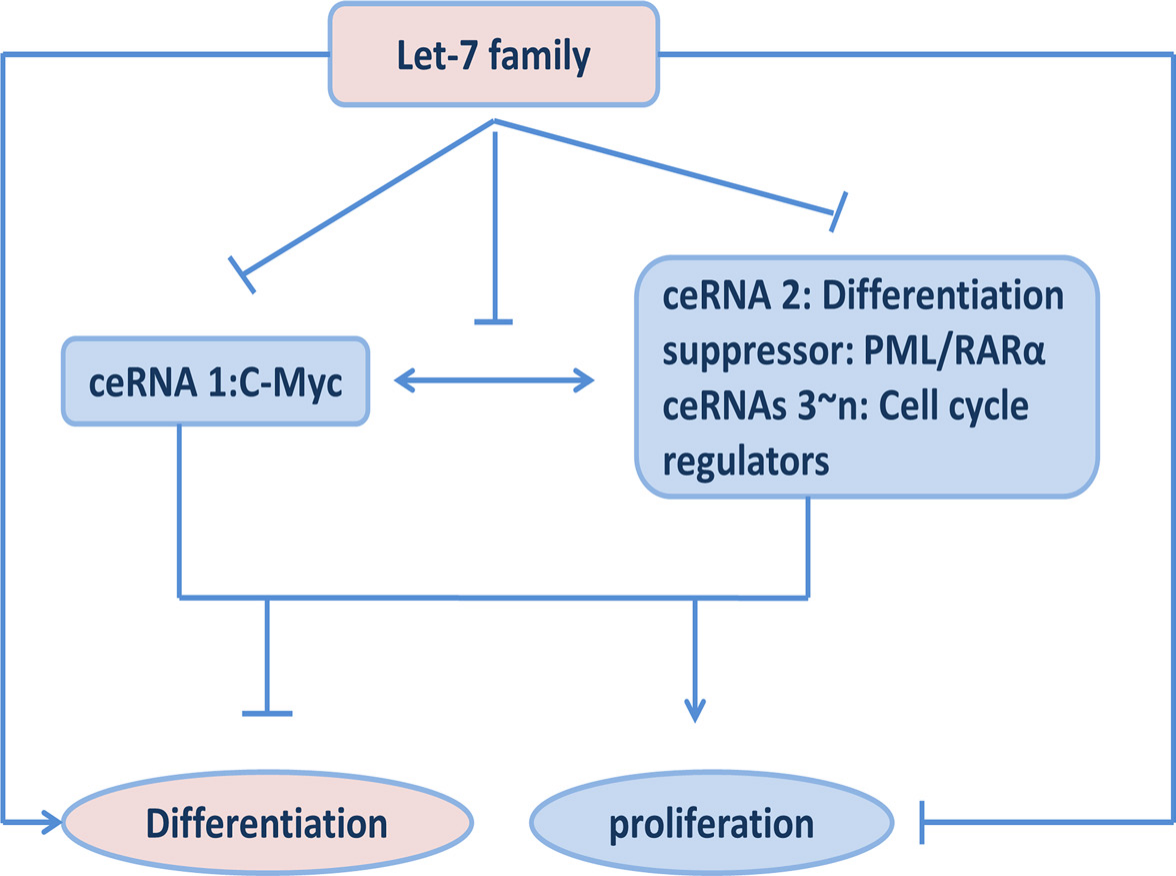
Effects of ceRNA functions among c-My, PML/RARα and let-7 miRNAs on cell proliferation and differentiation

C-Myc has been shown to play a pivotal role in cell-fate decisions, including proliferation, differentiation and apoptosis [38–40]. Enforced expression of c-Myc blocks the differentiation and promotes cell growth in most hematopoietic cells [15]. It has been reported c-Myc expression must be negatively regulate in order for myeloblasts to differentiation [14]. Our study would provide a novel notion to explain the differentiation role of Myc downregulation. Decreased c-Myc transcript suppresses a series of genes through shared microRNAs released from Myc 3′UTR. PML/RARα is one of them which acts as a differentiation suppressor and shared let- 7 binding-sites with Myc. Additionally, let-7 often targets to multiple oncogenes required for cell cycle exit and represses directly cell proliferation. Therefore, decreased c-myc is concomitant with suppression of PML/RARα as well as other cell cycle genes in a sequence-specific manner to counteract proliferation, and subsequently allows a cell to commit to a differentiation pathway (Figure 5).

In our study, we focused only on a single microRNA family in our study. In fact, all of shared microRNAs targeting both c-Myc and PML/RARα would potentially involve in ceRNA regulation, not only limited to let-7 family. These miRNAs should be identified. Furthermore, our findings are based on the constant concentration of let- 7. But the level of let-7 expression is actually variable to maintain homeostasis and metabolism in cells. Therefore, an extensive microRNA-mediated dynamic network of RNA-RNA interactions should be further clarified in the future study.

Chromosome translocations are frequently observed in multiple subtypes of leukemia, these translocations result in UTR swap and consequent alter expression of their UTRs as well as the introduction of novel fusion site. It would lead to disrupting the balances between cell differentiation and growth by altering the distribution of microRNA molecules on all of other ceRNAs [41, 42]. Our findings that c-Myc acts as a potential ceRNA for PML/RARα through competition for let-7 miRNAs provide the evidence to support the hypothesis that chromosomal translocation in PML/RARα-fusion-mediated leukemogenesis can contribute to a disturbance in ceRNA regulation and play an important role in leukemic initiation and progression.

## MATERIALS AND METHODS

### Cell cultures and differentiation/apoptosis induction

Human APL cell lines (NB4) were grown in RPMI 1640 medium (Gibco, Carlsbad, CA, USA) supplemented with 10% fetal bovine serum (Australian origin), 2 mM L-glutamine and 100 Ug/mL penicillin/streptomycin. Human embryonic kidney cell lines (293T) were maintained in Dulbecco modified Eagle medium (Gibco) with the same supplements. Both cell lines were incubated at 37°C in a humidified atmosphere of 5% CO2. For granulocytic differentiation, NB4 was treated with either DMSO (solvent for ATRA) or 1 μm ATRA (Sigma-Aldrich) for 24, 48, 72 and 96 hours respectively. For induction of apoptosis, NB4 was treated with or without 1 μm ATO (Sigma-Aldrich) for 48 h.

### Transfection experiments

Silencer^®^ Select Pre-Designed and Validated siRNAs targeting human c-Myc, Dicer1, non-targeting negative control and mirVana^®^ miRNA mimics for mature let-7a,7c, 7g and scramble negative control were purchased from Ambion (Ambion, Austin, TX, USA). For RNA transfection, NB4 cells were seeded in 12-well dishes at a density of 150,000 cells per well and transfected 24 hr later with 10 nM siRNAs or 20 nM miRNA mimics using Dharmafect 2 (Dharmacon, Lafayette, CO, USA) according to the manufacture's recommendations. For plasmid transfection, 293T were seeded 24 hr before transfection at a density of 120,000 cells/well in 12-well dishes. Co-transfection of siRNAs or miRNA mimics with 1 ug psiCHECK-2+PML/RARα3′UTR into 293T cells was performed with the transfection reagent, DharmaFECT Duo (Dharmacon), following the manufacturer's instructions.

### RNA extraction and quantitative real-time PCR

Total RNA containing miRNA was extracted using miRNeasy Mini Kit (Qiagen, Hilden, Germany) as per the manufacturer's instructions. For analysis of mRNAs, cDNA from mRNA was synthesized according to the protocol of RevertAid First Strand cDNA Synthesis Kit (Fermentas, Burlington, CA) and qPCR analysis was performed using FastStart Universal SYBR Green Master (Roche, Indianapolis, IN, USA) on a 7500 Real-Time PCR System (Applied Biosystems, Foster City, CA, USA).

The specific primer sets were:

c-Myc forward: 5′-GCTGCTTAGACGCTGGATTT TT-3′

c-Myc reverse: 5′-ACCGAGTCGTAGTCGAGGTC AT-3′

PML/RARα forward: 5′- AAGTGAGGTCTTCCTG CCCAA-3′

PML/RARα reverse: 5′-GGCTGGGCACTATCTCT TCAGA-3′

GAPDH forward: 5′-ACACCATGGGGAAGGTG AAG-3′

GAPDH reverse: 5′-AAGGGGTCATTGATGGCA AC-3′

The synthesis of cDNA from miRNA was prepared with miScript II RT Kit (Qiagen) according to the manufacturer's instructions. MiScript Primer Assays for let-7 and RNU6B (used as endogenous control and normalizer) are mature miRNA-specific primers (Qiagen) used in combination with the miScript SYBR Green PCR Kit (Qiagen) for real-time PCR of miRNA. The comparative Ct method was used to quantify target genes relative to the endogenous GAPDH or RNU6B control.

### Western blot analysis

The cells were harvested at 48 h after transfection and lysed by RIPA buffer for 30 min at 4°C. 25 μg proteins were resolved by SDS-PAGE on 10% gels. The following primary antibodies were used for endogenous protein western blots: c-Myc, total-AKT and β-actin (all from Cell Signaling Technology, Beverly, MA, USA) at 1:1000 dilutions, PML/RARα (Santa Cruz Biotechnology, Santa Cruz, CA) at 1:400 dilutions. Secondary HRP-tagged antibodies (Pierce, Rockford, IL, USA) were used at 1:20,000 dilutions.

### Cell proliferation assay

Cell proliferation analysis was performed using the Cell Counting Kit-8 CCK-8 (Tongren, China). Cells were plated in triplicates in 96-well plates at a final density of 20,000/well 10 hr posttransfection. At the point of 24, 48, and 72 h, 10 ul CCK-8 reagents were added to each well and further incubated for 2 h at 37°C. The number of viable cells was assessed by the optical density (OD) at 450 nm with a microplate spectrophotometer.

### FACS analysis

For analysis of the cell surface markers CD11b for granulocytic differentiation, NB4 cells were treated with DMSO or 1 μm ATRA 12 h after transfection with miRNA mimics, and the percentage of CD11b-positive cells was then determined 48 hr later using PE-conjugated mouse antihuman CD11b antibody (BD Pharmingen) via flow cytometry. Cell apoptosis was performed after 72 hr transfection using Annexin V-PE/7-AAD kit (BD PharMingen, San Diego, CA) according to manufacturer's protocol and subsequently analyzed by flow cytometry analysis on Flow Cytometry System (Becton Dickson, USA).

### Plasmids construction and luciferase assays

The 3′UTR of PML/RARα containing the predicted let-7-binding sites was amplified by PCR from the genomic DNA of NB4 cells and was inserted into the dual-luciferase psiCHECK-2 reporter vector using XhoI and NotI restriction sites.

Primer sequences were as follows:

PML/RARα3′UTR-F: GCCCACGCCACATGGA CAC,

PML/RARα3′UTR-R: CTCAGTGGAAAGCCCAA CAG

Luciferase activity was determined 24 hr post-transfection with the dual-luciferase reporter assay system (Promega, Madison, WI, USA) according to manufacturer's instructions. Firefly luciferase activity was then normalized with Renilla luciferase activities in the same well.

### Statistical analysis

All experiments were performed in triplicate at least three times and representative results are shown. Statistical significance was assessed by the Student's *t*-test. All data were presented as the mean ± SD. Values of *p* < 0.05 was considered to be statistically significant.

## Abbreviations

ceRNAs: competing endogenous RNA
APL: Acute Promyelocytic Leukemia
ATRA: All-Trans-Retinoic Acid
DMSO: Dimethyl Sulfoxide
ATO: Arsenic Trioxide
